# *C-erbB-2* or mutant *Ha-ras* induced malignant transformation of immortalized human ovarian surface epithelial cells *in vitro*

**DOI:** 10.1038/sj.bjc.6601423

**Published:** 2003-12-09

**Authors:** T Kusakari, M Kariya, M Mandai, Y Tsuruta, A A Hamid, K Fukuhara, K Nanbu, K Takakura, S Fujii

**Affiliations:** 1 Department of Gynecology and Obstetrics, Faculty of Medicine, Kyoto University, 54 Shogoin Kawahara-cho, Sakyo-ku, Kyoto, 606-5807, Japan

**Keywords:** ovarian surface epithelium, immortalization, malignant transformation, ovarian cancer

## Abstract

Ovarian cancer is believed to develop from the ovarian surface epithelium through the accumulation of aberrations of oncogenes and/or tumor suppressor genes. However, it is unclear how the gene abnormalities are involved in ovarian carcinogenesis. To elucidate the process, we transfected genes reported to show their abnormalities in human ovarian cancers into human ovarian surface epithelial cells. Immortalization of the cells was achieved by the transfection of SV40 large T antigen (LT) and human telomerase reverse transcriptase (hTERT); however, the resultant cells showed no tumorigenesis. Additional transfection of either *c-erbB-2* or mutant *Ha-ras* into the immortalized cells showed the anchorage-independent growth and tumorigenesis in mice with the incidence of 50% and 40%, respectively. Histologically, all the tumours were undifferentiated. In association with the tumorigenesis, the cells expressing *c-erbB-2* or mutant *Ha-ras* demonstrated increased vascular endothelial growth factor secretion under hypoxia and enhanced resistance to apoptosis compared with the immortalized cells. Collectively, the introduction of either *c-erbB-2* or mutant *Ha-ras* in the cells, which were efficiently immortalized by the transfection of LT and hTERT, showed tumorigenicity, suggesting that *c-erbB-2* or mutant *Ha-ras* genes might be involved in ovarian carcinogenesis.

Ovarian cancer is the leading cause of death in women with gynaecological malignant tumours (Herbst, 1994). Despite the recent developments in aggressive surgery and chemotherapy, the prognosis remains poor because ovarian cancers are often not diagnosed until advanced stages and the cancer cells frequently acquire drug resistance during repeated chemotherapies. Thus, there is an urgent need to develop new therapies. Epithelialovarian cancer is believed to develop from the ovarian surface epithelium (OSE) (Scully, 1977), through the accumulation of aberrations of oncogenes and/or tumour suppressor genes. Many gene abnormalities have been detected in ovarian cancers, for example, the overexpression of *c-erbB-2*, mutation of *Ki-ras*, *p53*, *BRCA1* or *BRCA2*, and the expression of telomerase activity (Matias-Guiu and Prat, 1998). Nonetheless, it is still unclear how the gene abnormalities are involved in the process of ovarian carcinogenesis.

Human OSE cells provide a useful *in vitro* culture system for the analysis of pathogenesis of ovarian cancers using molecular biology and genetic techniques. Two studies on malignant transformation of human OSE cells by gene transfection have been reported (Ong *et al*, 2000; Nitta *et al*, 2001). A recent report demonstrated that transfection of human telomerase reverse transcriptase (hTERT) into SV40 large T antigen (LT)-transfected human somatic cells showed 100% immortalization in transfected cells (Hahn *et al*, 1999). Therefore, in the present study, we tried to induce malignant transformation of cultured human OSE by transfecting genes that were shown to be altered in human ovarian cancers. We also tried to analyse the phenotypic change induced in the transformation process.

## MATERIALS AND METHODS

### Cell culture

Human OSE cells were obtained with written consent from patients who underwent abdominal surgery for benign gynaecological diseases. Ovarian surface epithelium cells were collected during surgery and cultured as previously reported (Kuroda *et al*, 2001). The purity of the cultured OSE cells was confirmed by positive immunostaining of both cytokeratin and vimentin with negative staining of factor-VIII-related antigen (Auersperg *et al*, 1994). The human ovarian cancer cell line A2780 was maintained as previously reported (Tsuruta *et al*, 2001). The fibroblast cell line NIH 3T3 cells were cultured in Dulbecco's modified Eagle's medium (DMEM) (Nissui, Tokyo, Japan) with 10% FCS.

### Gene transfection

Subconfluent cultures of OSE cells in passage 1 were transfected with 20 *μ*g of pSV3neo, which contains LT gene, using the Lipofectin reagent (Gibco, NY, USA). Next, we transfected 20 *μ*g of hTERT plasmid (pcDNA3-hTERTn2) into LT-transfected OSE cells. Cells expressing the hTERT gene were selected in media supplemented with 400 *μ*g ml^−1^ of G418 and were cloned. We finally transfected 20 *μ*g of the *c-erbB-2* plasmid (pSV2erbB2) or the mutant *Ha-ras* plasmid (pSNTT) into hTERT- and LT-transfected OSE cells with 5 *μ*g pHygEGFP vector (Clontech, CA, USA). We harvested those clones that fluoresced as successfully transfected cells.

### Western blotting

Western blotting was performed as previously described (Tsuruta *et al*, 2001). We used the following antibodies as a probe: anti-SV40 mouse monoclonal antibody (Santa Cruz, CA, USA), anti-*Ha-ras* mouse monoclonal antibody (Santa Cruz) and anti-*c-erbB-2* rabbit polyclonal antibody (Nichirei, Tokyo, Japan).

### RT–PCR analysis

The expression of hTERT mRNA and the telomerase activity were performed by RT–PCR and by stretch PCR assay, respectively, as previously reported (Kyo *et al*, 1999).

### Immunohistochemistry

Tumour tissues were fixed in 10% buffered formalin, embedded in paraffin and sectioned. Sections were stained with haematoxylin and eosin. For the immunohistochemical staining, they were deparaffinized in xylol for 30 min and processed as previously described (Kuroda *et al*, 2001). We used the following antibodies as primary antibodies: a rabbit polyclonal anti-cytokeratin antibody (Santa Cruz), a rabbit polyclonal anti-vimentin antibody (Santa Cruz), a mouse monoclonal anti-CA125 antibody (Dako, CA, USA) or a mouse monoclonal anti-SV40 large T antigen (Santa Cruz).

### Life span determinations

Cells were seeded on 35 mm collagen type-I-coated culture dishes. When confluent, the cells were trypsinized and seeded at 1 : 3 split ratio. This process was continued until the cells became stationary (senescence), that is did not become confluent after 1 month in culture. The population doublings (PDs) were added for each passage until the stationary phase was reached.

### Assay for anchorage independence

Colony formation in semisolid agar were assayed by suspending 10^4^ cells in 2 ml appropriate medium with 0.33% agarose (Gibco) and placing this suspension on the top of 5 ml solidified 0.5% agarose in the above medium. Triplicate cultures for each cell clone were maintained at 37°C in a 5% CO_2_ atmosphere. Colonies over 50 *μ*m in diameter were counted after 2 weeks. The experiments were repeated three times for each clone.

### Tumorigenicity assays

Female BALB/C nude mice, 4–6 weeks old, were purchased from CLEA JAPAN (Tokyo, Japan). The animals received proper care according to the rules of the Kyoto University Committee on Animal Care. Animal experiments were performed comply with the United Kingdom Co-ordinating Committee on Cancer Research (UKCCCR) Guidelines (Workman *et al*, 1988). Approximately 10^8^ cells per animal were injected subcutaneously in nude mice. If subcutaneous mass was confirmed, they were killed and the tumours resected. Cells were considered nontumorigenic if mouse failed to form tumour within 4 months of injection.

### Growth parameters

Population doubling time (PDT) was determined as described below. Cells (10^4^ cells well^−1^) were seeded in each well of a collagen type-I-coated 24-well plate. Duplicate cell counts were determined daily for 7 days using a cell counter (Coulter, FL, USA). The natural logarithm of the mean number of cells per dish was plotted as a function of time in hours. The maximum doubling time was calculated from the steepest part of the plot. The experiments were repeated three times for each clone.

### Measurement of bromodeoxyuridine (BrdU) incorporation rate by flow cytometry

The measurement of BrdU incorporation rate by flow cytometry was performed using the FITC-conjugated BrdU antibody (Anti-BrdU) (Becton-Dickinson, Bedford, MA, USA) according to the manufacturer's instructions. Briefly, 10^8^ cells of each sample were incubated with 10 *μ*M BrdU (Nacalai tesque, Kyoto, Japan) for 30 min and washed with PBS. The cells were collected with cell scraper, fixed in 70% ethanol and denatured with 2 N Hydrochloric acid/Triton X-100 to produce single-stranded molecules. They were resuspended in 0.1 M Sodium Tetraborate Decahydrate, pH 8.5 to neutralise the acid. Then, they were incubated with anti-BrdU (Becton-Dickinson) and were resuspended in a DNA-staining solution containing propidium iodine and RNase (Cycle TEST™ PLUS DNA Reagent Kit) (Becton-Dickinson). The rate of BrdU incorporation was determined by a FACS Caliber flow cytometer (Becton-Dickinson) and analysed with CELL Quest (Becton-Dickinson). The experiments were repeated three times.

### Measurement of apoptosis by ELISA using antihistone antibody

Apoptosis induced by serum deprivation was quantified by measurement of DNA fragmentation with Cell Death Detection ELISA (Roche, Mannheim, Germany) as previously described (Kuroda *et al*, 2001). Assays were performed in duplicate. Induced apoptosis was expressed as relative cell death, which was a ratio of apoptosis level detected in culture without serum to apoptosis level of control detected in culture with 15% serum. The experiments were repeated three times.

### Measurement of human vascular endothelial growth factor (VEGF) secretion by ELISA

The level of vascular endothelial growth factor (VEGF) in culture supernatants was measured by AN'ALYZA™ (TECHNE, MN, USA). Each assay was performed in duplicate. The cells were cultured on 60 mm collagen type-I-coated culture dishes. When confluent, all cultures were then maintained in medium with 15% FCS at 37°C under 20 or 1% O_2_ concentration for 72 h. Cells were collected with 2.5% trypsin and cell numbers were counted. The supernatants were collected and the amount of human VEGF in supernatants was measured with ELISA according to the manufacturer's protocol. The values of VEGF were standardised with cell number. The experiments were repeated three times.

### Statistical analysis

For analysis of between-group differences, *P*-values were determined using Mann–Whitney's *U*-test. A *P*-value less than 0.05 was considered significant.

## RESULTS

### Confirmation of introduced gene expression in OSE cells

**Figure 1:**
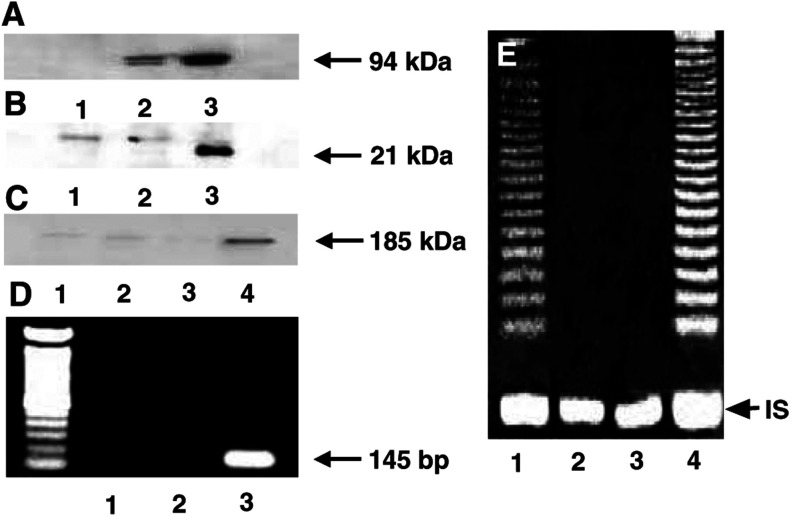
Protein expressions evaluated by Western blotting for LT (**A**), mutant *Ha-ras* (**B**) and *c-erbB-2* (**C**). The expression of hTERT mRNA and telomerase activity in OSE cells by RT-PCR, strech PCR, respectively (**D, E**). (**A**) Lane 1: OSE cells; lane 2: LT-transfected OSE cells; lane 3: OSE cells transfected with a combination of LT and hTERT. (**B**). Lane 1: OSE cells; lane 2: OSE cells transfected with a combination of LT and hTERT; lane 3: OSE cells transfected with a combination of LT, hTERT and mutant *Ha-ras*. (**C**) Lane 1: OSE cells; lane 2: LT-transfected OSE cells; lane 3: OSE cells transfected with a combination of LT and hTERT; lane 4: OSE cells transfected with a combination of LT, hTERT and *c-erbB-2*. (**D**) Lane 1: OSE cells; lane 2: LT-transfected OSE cells; lane 3: OSE cells transfected with a combination of LT and hTERT. (**E**) Lane 1: positive control; lane 2: negative control; lane 3: LT-transfected OSE cells; lane 4: OSE cells transfected with a combination of LT and hTERT. IS=internal standard.

To confirm the successful expression of introduced gene products in OSE cells, the expressions of LT, *c-erbB-2* and mutant *Ha-ras* protein were confirmed by Western blotting, as shown in Figure 1A, B and C

, respectively. The hTERT mRNA expression and telomerase activity in hTERT-introduced OSE cells were confirmed by RT–PCR (Figure 1D) and by stretch PCR assay (Figure 1E), respectively.

### Malignant transformation of cultured OSE cells

To evaluate malignant tranformation of OSE cells transfected with various genes, we analysed immortalization, ability of anchorage-independent growth and tumorigenicity in mice. Untransfected OSE cells underwent senescence in 3±1 passages in culture. Transfection with LT extended the life span of OSE cells, but, these LT-transfected OSE cells (OSE+LT) underwent senescence in 9±2 passages. In contrast, the PDs of OSE cells transfected with a combination of LT and hTERT (OSE+LT+hTERT) was 116±11 passages and the cells did not achieve senescence, suggesting that they had been immortalized. Ovarian surface epithelium cells transfected with a combination of LT, hTERT and mutant *Ha-ras* (OSE+LT+hTERT+*Ha-ras*), and OSE cells transfected with a combination of LT, hTERT and *c-erbB-2* (OSE+LT+hTERT+*c-erbB-2*) clones also were proliferating without senescence with PDs of 106±11 and 106±11 passages to date, respectively. As for growth ability in soft agar, the results of the assay for anchorage-independent growth are shown in Table 1

**Table 1:** Anchorage-independent growth of primary OSE, gene-transfected OSE cells and ovarian cancer cell line

**Cell groups**	**The number of colonies**
OSE cells	1.2±0.8
OSE cells+LT	6.0±1.5^a^
OSE cells+LT+hTERT	6.0±2.1^a^
OSE cells+LT+hTERT+*Ha-ras*	21.8±3.3^b^
OSE cells+LT+hTERT+*c-erbB-2*	43.4±5.0^c^
A2780	398.4±27.7^d^

The values are means±s.d. of three clones, OSE cells+LT=LT-transfected OSE cells; OSE cells+LT+hTERT=OSE cells transfected with a combination of LT and hTERT; OSE cells+LT+hTERT+*Ha-ras*=OSE cells transfected with a combination of LT, hTERT and mutant *Ha-ras*. OSE cells+LT+hTERT+*c-erbB-2*=OSE cells transfected with a combinationof LT, hTERT and *c-erbB-2*; A2780=ovarian cancer cell line.

a *P*<0.05 against OSE cells.

b *P*<0.05 against OSE cells+LT+hTERT.

c *P*<0.05 against OSE cells+LT+hTERT+*Ha-ras*.

d *P*<0.05 against OSE cells+LT+hTERT+*c-erbB-2*.

.

OSE+LT and OSE+LT+hTERT showed slightly enhanced proliferative activity in soft agar compared with original OSE cells. The additional introduction of *c-erbB-2* or mutant *Ha-ras* into OSE+LT+hTERT cells further enhanced the growth activity with statistical significance (*P*<0.05). OSE+LT+hTERT+*c*-*erbB-2* cells showed significantly enhanced growth activity in soft agar compared with that of OSE+LT+hTERT+*Ha-ras* cells (*P*<0.05). However, compared with the A2780 ovarian cancer cell line, colonies of OSE+LT+hTERT+*c-erbB-2* cells were small in number. We examined tumorigenicity in immunodeficient mice. As shown in Table 2

**Table 2:** Tumour formation incidence of OSE cells and gene-transfected OSE cells injected into nude mice

**Groups**	**BALB/C nude mice**
OSE cells	0/10(0%)
OSE cells+LT	0/10(0%)
OSE cells+LT+hTERT	0/10(0%)
OSE cells+LT+hTERT+*Ha-ras*	4/10(40%)
OSE cells+LT+hTERT+*c-erbB-2*	5/10(50%)
A2780	10/10(100%)

Cells were subcutaneously injected into nude mice. The results are presented as number of mice with tumour formation/total number of mice injected with OSE cells with or without gene transfection, with this value expressed as a percentage in parentheses. OSE cells+LT=LT-transfected OSE cells; OSE cells+LT+hTERT=OSE cells transfected with a combination of LT and hTERT. OSE cells+LT+hTERT+*Ha-ras*=OSE cells transfected with a combination of LT, hTERT and mutant *Ha-ras*; OSE cells+LT+hTERT+*c-erbB-2*=OSE cells transfected with a combination of LT, hTERT and *c-erbB-2*; A2780=ovarian cancer cell line.

, the OSE cells expressing LT or both LT and hTERT did not form tumours. In contrast, those cells additionally transfected with *c-erbB-2* and mutant *Ha-ras* formed tumours in five of 10 cases (50%), four of 10 cases (40%), respectively, in nude mice within 1–3 months. On the other hand, injection of A2780 in nude mice formed tumour in 10 of 10 cases (100%) within 2–3 weeks.

### Morphologic features of tumours formed in mice

**Figure 2:**
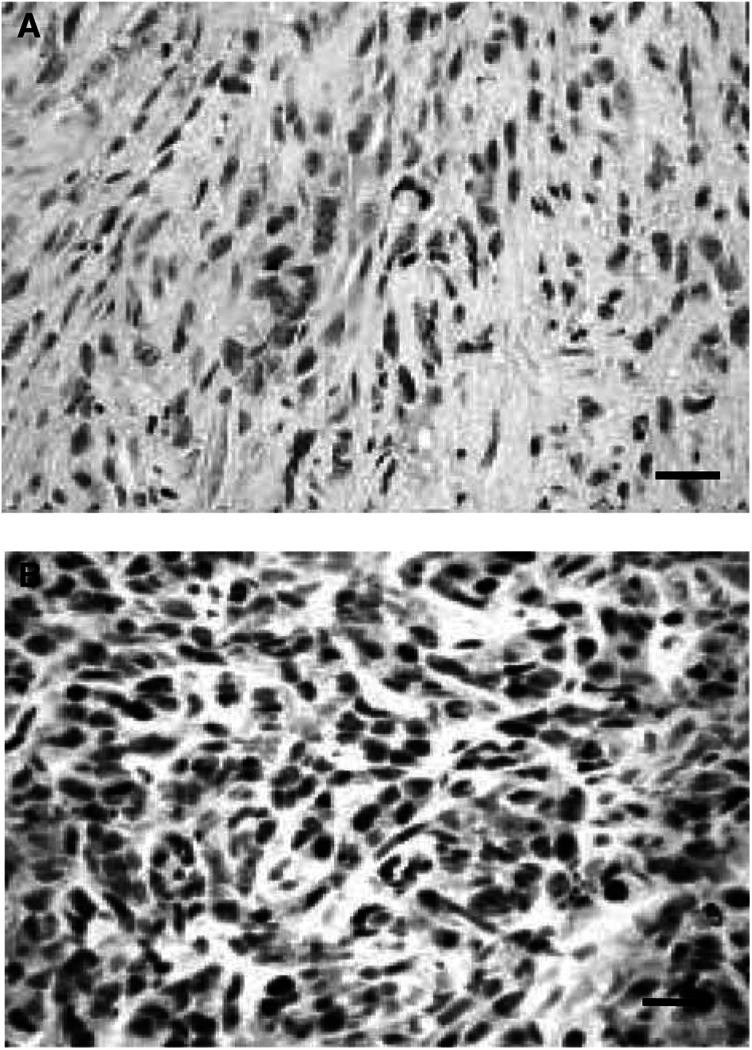
Microscopic and immunohistochemical findings of tumours formed in mice. (**A**) Microscopic findings of tumours expressing mutant *Ha-ras.* The tumours, irrespective of the introduced gene, *c-erbB-2* or mutant *Ha-ras*, were shown to be composed of spindle-shaped cells with minimal nuclear atypia and less frequent mitoses. (**B**) Immunohistochemistry of tumour cells transfected with a combination of LT, hTERT and mutant *Ha-ras*, using the anti-SV40 large T antigen antibody. All tumour cells were positively stained for the antigen, indicating that these tumours originate from the injected transformed human OSE cells. Scale bar, 200 *μ*m.

Morphologic features of inoculated tumours were assessed by microscopic observation. Histologically, all the tumours expressing mutant *Ha-ras* were made up in major part of homogenous spindle-shaped cells with minimal nuclear atypia and infrequent mitoses (Figure 2A

) Immunohistochemically, tumours were positive for cytokeratin and negative for vimentin, suggesting that they were epithelial tumours. All the tumours formed by the injection of *c-erbB-2*-expressing cells showed the same features as described above, except that they were positive for vimentin. All the tumours were confirmed to be derived from the injected OSE cells by immunohistochemical expression of LT protein (Figure 2B).

### Effect of gene transfections on proliferative activity

To investigate whether introduced genes have an effect on proliferation activity in OSE cells during the malignant transformation, PDT measurement and BrdU incorporation assay were performed. LT-transfected OSE cells showed approximately one third the PDT of the original OSE cells, indicating that the proliferative activity accelerated. Moreover, OSE+LT+hTERT showed a significant decrease in PDT compared with OSE+LT. However, there were no significant decreases in PDT associated with the introduction of *c-erbB-2* or mutant *Ha-ras* into OSE+LT+hTERT cells (Table 3

**Table 3:** Population doubling time in primary OSE and gene-transfected OSE cells

**Cell groups**	**Population doubling time(h)**
OSE cells	97.4±11.7
OSE cells+LT	30.7±2.3^a^
OSE cells+LT+hTERT	7.4±0.7^b^
OSE cells+LT+hTERT+*Ha-ras*	7.3±1.3
OSE cells+LT+hTERT+*c-erbB-2*	7.4±0.8

The values are means±s.d. of three clones studied. OSE cells+LT=LT-transfected OSE cells; OSE cells+LT+hTERT=OSE cells transfected with a combination of LT and hTERT; OSE cells+LT+hTERT+*Ha-ras*=OSE cells transfected with a combination of LT, hTERT and mutant *Ha-ras*; OSE cells+LT+hTERT+*c-erbB-2*=OSE cells transfected with a combination of LT, hTERT and *c-erbB-2*.

a *P*<0.05 against OSE cells.

b *P*<0.05 against OSE cells+LT.

). By a BrdU incorporation assay, the populations at the S phase of the total population in OSE+LT+hTERT were significantly increased compared with that of NIH 3T3, which has normal cell cycle (45.6±3.6, 1.6±1.4% respectively) (*P*<0.05). The population rate at S phase in OSE+LT+hTERT+*Ha-ras* and OSE+LT+hTERT+*c-erbB-2* were not significantly different (45.3±5.3% and 42.6±6.9% respectively), consistent with the results of PDT (Table 4

**Table 4:** Population rate at S phase of cell cycle in gene-transfected OSE cells

**Cell groups**	**Population rate at S phase (%)**
NIH 3T3	1.6±1.4
OSE cells+LT+hTERT	45.6±3.6^a^
OSE cells+LT+hTERT+*Ha-ras*	45.3±5.3
OSE cells+LT+hTERT+*c-erbB-2*	42.6±6.9

The values are means±s.d. of three clones studied. The results are presented as population rate at S phase of cell cycle as percentage . NIH 3T3=fibroblast cell line; OSE cells+LT+hTERT=OSE cells transfected with a combination of LT and hTERT; OSE cells+LT+hTERT+*Ha-ras*=OSE cells transfected with a combination of LT, hTERT and mutant *Ha-ras*; OSE cells+LT+hTERT+*c-erbB-2*=OSE cells transfected with a combination of LT, hTERT and *c-erbB-2*.

a *P*<0.05 against NIH 3T3.

).

### Effect of the genes on apoptosis resistance

**Figure 3:**
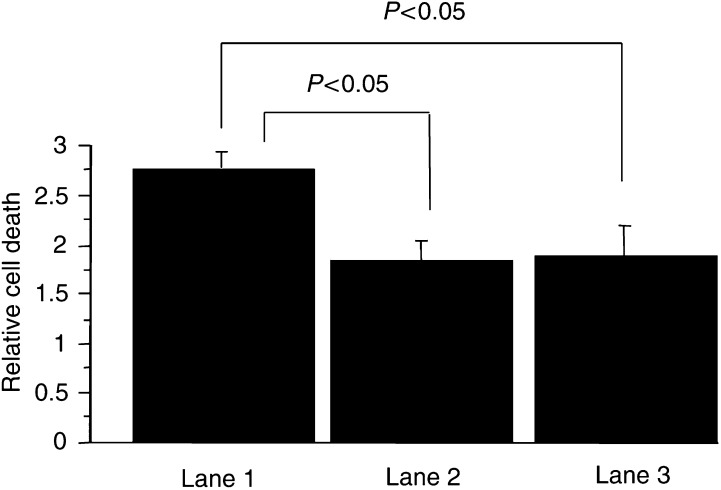
Levels of apoptosis induced by serum deprivation in the immortalised and tumorigenic OSE clones, which expressed as relative cell death. Relative cell death was a ratio of apoptosis level induced by culture without serum to apoptosis level of control detected in culture with 15% serum. The tumorigenic OSE cells expressing *c-erbB-2* or mutant *Ha-ras* demonstrated the lower level of apoptosis compared with the immortalized OSE cells (*P*<0.05). Lane 1: OSE cells transfected with a combination of LT and hTERT, lane 2: OSE cells transfected with a combination of LT, hTERT and *c-erbB-2*, lane 3: OSE cells transfected with a combination of LT, hTERT and mutant *Ha-ras*.

To investigate the detail mechanisms of how *c-erbB-2* or mutant *Ha-ras* genes are involved in the malignant transformation of OSE cells, the effect of these genes on apoptosis resistance was evaluated. Apoptosis was induced by serum deprivation for 72 h. The level of apoptosis in tumorigenic *c-erbB-2*- or mutant *Ha-ras*-expressing OSE cells was measured by ELISA and compared with that of the nontumorigenic OSE cells. Tumorigenic OSE+LT+hTERT+*c-erbB-2* or mutant *Ha-ras* demonstrated the lower level of apoptosis, compared with immortalised OSE+LT+hTERT cells (*P*<0.05), demonstrating that the tumorigenic OSE cells possess enhanced resistance to apoptosis than the immortalized, but nontumorigenic OSE cells (Figure 3

).

### Effect of the genes on VEGF secretion

**Figure 4:**
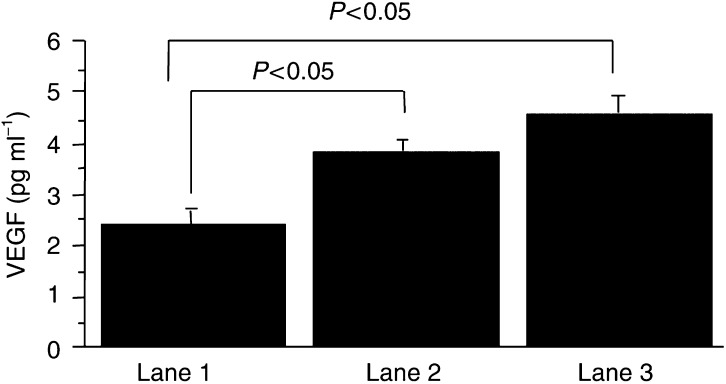
Amount of human VEGF in culture supernatants of the immortalized and tumorigenic OSE clones. Cells were cultured under hypoxic condition of 1% O_2_, and VEGF level for tumorigenic OSE cells transfected with a combination of LT, hTERT and *c-erbB-2* or mutant *Ha-ras* was significantly higher than that for immortalized OSE cells (*P*<0.05). Lane 1: OSE cells transfected with a combination of LT and hTERT, lane 2: OSE cells transfected with a combination of LT, hTERT and *c-erbB-2*, lane 3: OSE cells transfected with a combination of LT, hTERT and mutant *Ha-ras*.

We also analysed whether *c-erbB-2* or mutant *Ha-ras* genes have effect on upregulation of VEGF secretion. When cells were cultured under hypoxic condition, VEGF level in supernatant, measured by ELISA, significantly increased, compared with that under room air for each immortalized or tumorigenic OSE group. As shown in Figure 4

, in the hypoxic condition, VEGF level for tumorigenic OSE+LT+hTERT+*c-erbB-2* or mutant *Ha-ras* was significantly higher than that for immortalized OSE+LT+hTERT cells (*P*<0.05).

## DISCUSSION

Epithelial ovarian cancer is believed to develop from the OSE (Scully, 1977), through the amplifications of oncogenes and mutations of tumour suppressor genes. Although many gene abnormalities have been detected in ovarian cancers (Matias-Guiu and Prat, 1998), their roles in the process of ovarian carcinogenesis have not yet been elucidated. Human OSE cells provide a useful experimental system for the analysis of ovarian carcinogenesis. Two studies on immortalization of human OSE cells by the introduction of LT alone have been already reported (Maines-Bandiera *et al*, 1992; Nitta *et al*, 2001). Maines-Bandiera *et al* reported that OSE lines transfected with LT exhibited an extended life span, but subsequently underwent a progressive reduction of growth and characteristic morphologic changes associated with senescence within 20 passages. Nitta *et al* reported that only six out of 10 OSE lines transfected with LT were immortalized, and they had very high telomerase activity. These results suggest that introduction of LT alone is not sufficient to acquire unlimited growth capacity, and additional genetic change, that is telomerase activation, is necessary to accomplish immortalization. It is also reported that the immortalization of somatic cells by the introduction of LT alone is very infrequent (Van Der Haegen and Shay, 1993). On the other hand, a recent report showed that the transfection of hTERT into LT-transfected human somatic cells immortalized with the incidence of 100% (Hahn *et al*, 1999). In the present study, we tried to immortalize cultured human OSE by the introduction of hTERT along with LT. As a result, PDs of these clones had already exceeded 100 without reaching senescence.

Immunohistochemically, the immortalized OSE cells expressing LT and hTERT were positive for cytokeratin, vimentin, but negative for CA125, and they were similar to the OSE cells of the primary culture (data not shown). Therefore, the immortalized OSE cells seem to maintain phenotypes similar to those of the original OSE cells, although the immortalized cells showed slight enhancement of growth ability in soft agar without tumorigenesis in mice. Thus, immortalized OSE cells expressing LT and hTERT are considered to be useful for the study of the oncogenic process of human ovarian cancers.

We selected *c-erbB-2* and mutant *Ha-ras* for the additional gene transfection for the following reasons. Amplification and/or overexpression of the *c-erbB-2* gene have been reported in 10–50% of all human epithelial ovarian cancers (Matias-Guiu and Prat, 1998). The *ras* oncoprotein consists of three subtypes, that is *Ha-ras*, *Ki-ras* and *N-ras*, all of which are thought to possess identical functions. The activation of *Ha-ras* by point mutation has been reported to be important for the genesis and maintenance of solid tumours (Chin *et al*, 1999). The *Ki-ras* mutation has been implicated in the pathogenesis of ovarian cancers (Matias-Guiu and Prat, 1998).

The additional introduction of *c-erbB-2* or mutant *Ha-ras* in immortalized OSE cells triggered tumorigenesis in mice. However, the incidence of tumour formation was 50% and 40% in cases of *c-erbB-2* and mutant *Ha-ras* introduction, respectively. The low incidence of tumour formation in our experiments cannot exclude the possibility that some unknown incidental genetic changes may be associated, especially because of p53 suppression by LT in the OSE cells. However, this possibility seems unlikely because all four tumours expressing mutant *Ha-ras* formed in the four different mice had similar histology with the same results of the immunohistochemical study. The consistency of histological features among the four different tumours strongly suggests that the malignant transformation was induced by the transfection of mutant *Ha-ras*. Five cases with tumours expressing *c-erbB-2* also showed consistency in the phenotypic features.

The growth activity of immortalized OSE cells expressing either *c-erbB-2* or mutant *Ha-ras* in soft agar were significantly lower when compared with that of the ovarian cancer cell line A2780, which showed tumorigenesis with 100% incidence. The latent period of tumour formation after injection of the former was 1–3 months, but that of the latter was 2–3 weeks. These results suggest that the malignant potential of immortalized OSE cells expressing either *c-erbB-2* or mutant *Ha-ras* may be lower than that of A2780. The low malignant potential of immortalized OSE cells expressing either *c-erbB-2* or mutant *Ha-ras* might be one of the causes of the low incidence of tumour formation in this study. Nonetheless, the loss of transfected genes after introduction by lipofection, or the varied efficiency of the post-transcriptional process of introduced genes may be the cause of the low incidence of tumor formation in this study. This seems unlikely because the immortalized cells expressing either *c-erbB-2* or mutant *Ha-ras* showed extension of their lifespan with stable growth activity, and because none of the clones showed significant deviation in the results of anchorage-independent growth and PDT assays. Therefore, we concluded that the additional introduction of *c-erbB-2* or mutant *Ha-ras* genes in the immortalized OSE cells was the cause of malignant transformation of human OSE cells, and these oncogenes might be playing an important role during the malignant transformation of OSE cells *in vivo*.

To clarify the mechanisms underlying introduction of *c-erbB-2* or mutant *Ha-ras* genetic material into the OSE cells and the resultant malignant transformation, we compared OSE cells before and after transfection of *c-erbB-2* or mutant *Ha-ras.* Interestingly, both the doubling times and the population rate at the S phase of the immortalized cells before the additional gene transfections and the tumorigenic cells expressing either *c-erbB-2* or mutant *Ha-ras* were nearly identical. Such a result suggests that the malignant transformation induced by introduction of *c-erbB-2* or mutant *Ha-ras* might proceed via signalling pathways unrelated to cell-cycle regulation. Resistance to apoptosis is considered to be one of the mechanisms underlying carcinogenesis, and genetic abnormalities of molecules regulating apoptosis have been detected in ovarian cancers (Matias-Guiu and Prat, 1998). The tumorigenic OSE cells expressing either mutant *Ha-ras* or *c-erbB-2* demonstrated the enhanced resistance to apoptosis induced by serum-deprivation, compared with the immortalized OSE cells. Neovascularisation is also critical in tumour formation and VEGF is a major angiogenic factor. The tumorigenic OSE cells expressing either *c-erbB-2* or mutant *Ha-ras* demonstrated the increased secretion of VEGF in hypoxic condition compared with the immortalized OSE cells. Such a finding suggests that tumorigenic cells might possess a greater capacity for neovascularisation. Reportedly, *ras* and *c-erbB-2* genes actually demonstrated angiogenic function, upregulating the expression of VEGF in colon cancer and in head and neck squamous cell carcinomas, respectively (O-charoenrat *et al*, 2000; Zhang *et al*, 2001). Taken together, *c-erbB-2* or mutant *Ha-ras* might allow malignant transformation of OSE cells via induction of resistance to apoptosis and enhancement of VEGF secretion.

In conclusion, immortalization of human OSE cells is induced efficiently by the transfection of hTERT in addition to LT. Although the incidence is not so high, introduction of either *c-erbB-2* or mutant *Ha-ras* into immortalized cells results in formation of malignant tumours. Further study is necessary to elucidate the genes involved in the oncogenesis of human ovarian cancer using this kind of immortalized OSE cells.
